# A Microfluidic Chamber for Analysis of Neuron-to-Cell Spread and Axonal Transport of an Alpha-Herpesvirus

**DOI:** 10.1371/journal.pone.0002382

**Published:** 2008-06-18

**Authors:** Wendy W. Liu, Joseph Goodhouse, Noo Li Jeon, L. W. Enquist

**Affiliations:** 1 Department of Molecular Biology, Princeton University, Princeton, New Jersey, United States of America; 2 Department of Biomedical Engineering, University of California Irvine, Irvine, California, United States of America; Yale University, United States of America

## Abstract

Alpha-herpesviruses, including herpes simplex virus and pseudorabies virus (PRV), infect the peripheral nervous system (PNS) of their hosts. Here, we describe an *in vitro* method for studying neuron-to-cell spread of infection as well as viral transport in axons. The method centers on a novel microfluidic chamber system that directs growth of axons into a fluidically isolated environment. The system uses substantially smaller amounts of virus inoculum and media than previous chamber systems and yet offers the flexibility of applying multiple virology and cell biology assays including live-cell optical imaging. Using PRV infection of cultured PNS neurons, we demonstrate that the microfluidic chamber recapitulates all known facets of neuron-to-cell spread demonstrated in animals and other compartmented cell systems.

## Introduction

An intriguing aspect of the alpha-herpesvirus life cycle in the natural host is the controlled spread of virus within the peripheral nervous system (PNS), with rare invasions of the central nervous system. After establishing a primary infection at peripheral epithelial tissues, alpha-herpesviruses, such as herpes simplex virus and pseudorabies virus (PRV), invade the termini of sensory and motor neurons that innervate the infected site. Following transport to the cell body through axons (retrograde transport), the viral genome usually remains latent in the nucleus. Months or years later, the latent infection can be reactivated, producing newly assembled viral particles. The viral particles then travel back to the periphery (anterograde transport), where re-infection of the epithelia produces infectious virus that can spread to naive hosts [1]. This long-distance movement of viral particles to and from the cell body engages microtubule-based fast axonal transport machinery [2]–[5].

The transport and targeting mechanisms behind the directional spread of infection between non-neuronal epithelial cells and neurons are poorly understood. This is in part due to the lack of facile *in vitro* systems that can model the complex biology of inter-cellular viral transmission. Earlier studies used compartmentalized Campenot chambers to study alpha-herpesvirus spread [6]–[15]. These chambers use a Teflon divider sealed to the surface of a tissue culture dish via a silicone grease layer. Neuronal cell bodies plated in one compartment extended axons to another compartment by penetrating the silicone grease. More recently, a chamber system that utilizes a Teflon ring placed on top of axons has also been developed [16]. These chambers can be challenging to assemble because slight mechanical disturbances or displacement of the grease seal can easily cause leakage or severing of neurites. Moreover, some chamber assemblies were not compatible with sophisticated microscopy techniques including live-cell imaging.

Microfluidic systems have been increasingly used by cell biologists to precisely control and manipulate cellular microenvironments [17]–[19]. Microfluidic devices fabricated with poly(dimethylsiloxane) (PDMS), a transparent and biocompatible polymer [20], [21], have been used for studies of cell migration [22]–[26], stem cells [27], and neurons [28], [29]. Here, we describe the use of a microfluidic chamber for culturing PNS neurons that enables us to analyze neuron-to-cell spread of alpha-herpesviruses by live-cell imaging as well as standard virology and cell biology methods.

**Figure 1:**
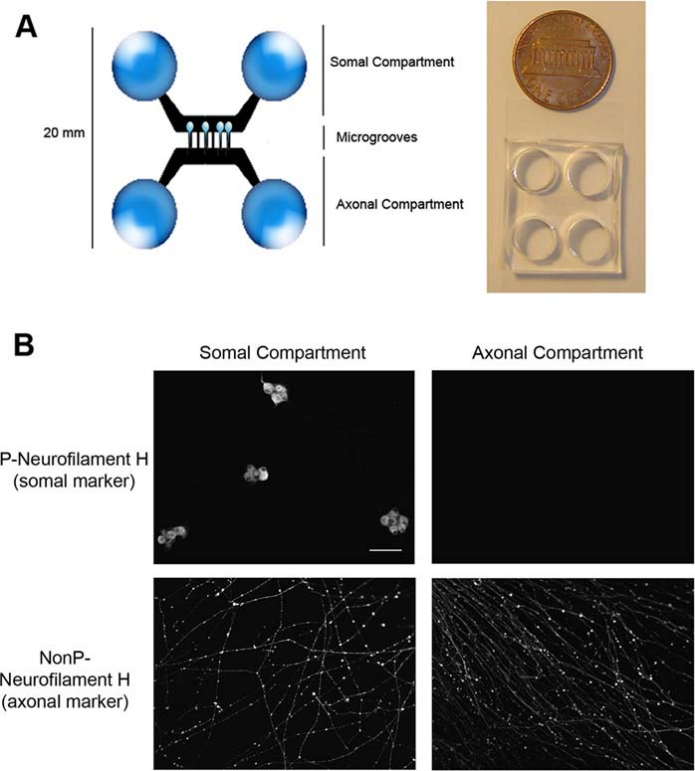
The microfluidic chamber system. (A) The chamber consists of a poly(dimethylsiloxane) (PDMS) mold containing mirror image compartments connected by microgrooves (450 μm in length, 10 μm in width). The PDMS adheres to a coated glass coverslip. Rat superior cervical ganglia (SCG) neurons are plated in the somal compartment and axon growth is guided into the axonal compartment through the microgrooves. (B) SCG neurons cultured in the chamber are polarized and mature after 9 d. Confocal microscopy images show neurons in the chamber fixed and stained for phosphorylated (P) neurofilament H, a somatodendritic marker, and nonphosphoylated (NonP) neurofilament H, an axon-specific marker. Bar: 150 μm.

The microfluidic device consists of a small PDMS piece with compartments connected by embedded microgrooves [30]. The whole device is placed against a glass coverslip and holds about 600 μl of media. Neuron cell bodies are cultured in the somal compartment, while axons extend through 10 μm wide microgrooves into the axonal compartment, where detector cells can be grown (Fig. 1A). A hydrostatic pressure difference between chambers is sufficient to isolate virions to either the somal or axonal compartment. Using the microfluidic chamber to study neuron-to-cell spread and axonal transport of PRV, we established several key parameters. First, plating non-neuronal epithelial cells in the axonal compartment enables neuron-to-cell spread of infection that is dependent on intact axons. Second, the attenuated vaccine strain PRV Bartha is defective in neuron-to-cell spread. Third, neuron-to-cell spread requires gB, a viral glycoprotein that mediates membrane fusion. However, it does not require gD, a glycoprotein necessary for infection by extracellular virions. Fourth, neuron-to-cell spread requires Us9, a viral membrane protein necessary for axonal sorting of viral structural proteins. Fifth, PRV Bartha has a slight defect in retrograde spread to cell bodies after entry at axons. Finally, the chamber can be used to image viral protein dynamics in live infected neurons. By imaging fluorescently tagged virion components, we find that fluorescent capsid structures associate with the outer tegument during anterograde transport in axons. In contrast, capsids lacking outer tegument traveled in the retrograde direction. Finally, we show that the velocity distribution of light particles lacking capsids is identical to that of capsids during anterograde transport. Hence, the microfluidic chamber recapitulates faithfully what has previously been observed in animal models and *in vitro* systems.

## Methods

### Cell and virus strains

The swine kidney epithelial cells (PK15) were cultured in Dulbecco's modified Eagle medium supplemented with 10% of fetal bovine serum and 1% penicillin/streptomycin. All PRV strains were propagated in PK15 cells. PRV strains used include: PRV Becker, a virulent isolate [31]; PRV Bartha, an attenuated vaccine strain [32]; PRV151, PRV Becker expressing green fluorescent protein (GFP) [33]; PRV152, PRV Bartha expressing GFP [34]; PRV180, expressing monomeric red fluorescent protein (mRFP) fused to the capsid protein VP26 [35]; and PRV181, expressing both GFP-VP22 and mRFP-VP26 fusion proteins [35]. PRV mutants in the PRV Becker background are PRV 160 (Us9 null) [36]; PRV GS442 (gD null mutant with gD replaced by the GFP open reading frame; provided by G. Smith, Northwestern University), which was grown on a gD complementing cell line [37]; and PRV HF22A (gB null mutant), which was grown on a gB complementing cell line (LP64e3).

### Neuronal cultures

Protocols for dissecting and culturing neurons are found in Ch'ng et al. [38]. Briefly, sympathetic neurons from the superior cervical ganglia (SCG) were dissected from E15.5 to E16.5 pregnant Sprague-Dawley rats (Hilltop Labs Inc., Pennsylvania) and incubated in 250 μg/ml of trypsin (Worthington Biochemicals) for 10 min. 1 mg/ml trypsin inhibitor (Sigma Aldrich) was applied to neutralize the trypsin for 5 min, after which it was removed and replaced with neuron culture medium. Prior to plating, the ganglia were dissociated using a fire-polished Pasteur pipette. The neurons were plated in the somal compartment of the microfluidic chamber placed on a glass coverslip that was previously coated with 500 μg/ml of poly-dl-ornithine (Sigma Aldrich) diluted in borate buffer and 10 μg/ml of natural mouse laminin (Invitrogen). The neuron culture medium consists of Dulbecco's modified Eagle medium (Invitrogen) and Ham's F12 (Invitrogen) in a 1∶1 ratio. The serum-free medium was supplemented with 10 mg/ml of bovine serum albumin (Sigma Aldrich), 4.6 mg/ml glucose (J. T. Baker), 100 μg/ml of holotransferrin (Sigma Aldrich), 16 μg/ml of putrescine (Sigma Aldrich), 10 μg/ml of insulin (Sigma Aldrich), 2 mM of l-glutamine (Invitrogen); 50 μg/ml or units of penicillin and streptomycin (Invitrogen), 30 nM of selenium (Sigma Aldrich); 20 nM of progesterone (Sigma Aldrich) and 100 ng/ml of nerve growth factor 2.5S (Invitrogen). The neuronal cultures are treated with 1 μM of cytosine β-d-arabinofuranoside (Sigma-Aldrich) to eliminate any non-neuronal cells two days after plating. The neuron culture medium was replaced every three days and cultures were kept in a humidified, CO_2_ regulated 37°C incubator. All experimental protocols related to animal use have been approved by The Institutional Animal Care and Use Committee of the Princeton University Research Board under protocol number 1543 in accordance with the regulations of the American Association for Accreditation of Laboratory Animal Care and those in the Animal Welfare Act (Public Law 99–198).

### Antibodies and fluorescent dyes

The primary antibodies used in this study include mouse monoclonal antiserum against the PRV major capsid protein VP5 (made by Alex Flood at the Princeton Monoclonal Antibody Facility [used at 1∶100]), rabbit antiserum against phosphorylated neurofilament H (SMI-31; Abcam [used at 1∶400]), and nonphosphorylated neurofilament H (SMI-32; Abcam [used at 1∶400]). All secondary Alexa fluorophores and the Hoechst nuclear dye were purchased from Molecular Probes.

### Microfluidic chamber system

The microfluidic chambers were fabricated as described in [30] and protocols for assembling the chambers were modified from previous reports [9], [30]. Briefly, glass coverslips were coated with 500 μg/ml of poly-dl-ornithine (Sigma). The chamber was then placed on the coated glass coverslips until it sealed to the glass around the chamber. The compartments were loaded with 10 μg/ml of natural mouse laminin (Invitrogen), incubated overnight and then washed and dried briefly. Neuronal medium was added to both compartments 24 h prior to plating the neurons. Each compartment holds about 300 μl of medium. After the SCG neurons were dissected and dissociated, approximately one half of a single ganglion (about 5,000 to 6,000 cell bodies) was plated into the two wells of one compartment, which we call the somal compartment (Fig. 1A). Neuron cultures were maintained as described above. All chambers have a microgroove length of 450 μm and a width of 10 μm.

### Assaying for neuron-to-cell spread of infection

Neurons were cultured for two weeks in the microfluidic chamber before manipulation. Two weeks postplating, after axons have extended across the microgrooves, PK15 cells were plated in the axonal compartment in neuronal medium supplemented with 1% fetal bovine serum. The cells were left to attach and contact the axons for at least 24 h. 330 μl of neuron medium was placed in the axonal compartment, after which 270 μl of viral inoculum was added to the somal compartment. This hydrostatic pressure difference completely prevented diffusion of infectious virions through the microgrooves. The neuronal cell bodies in the somal compartment were infected with sufficient virus to infect essentially all cells (around 10^6^ pfu diluted in neuronal medium). Direct calculations show that the multiplicity of infection (MOI) is around 100. After 1 hour, the viral inoculum was removed and replaced with 270 μl of neuron medium, with the volume difference between the two compartments maintained. The chambers were incubated in a humidified 37°C incubator until the appropriate time prior to imaging or titer determination. To determine the titer, PK15 cells in the axonal compartment were gently scraped from the dish with a pipette tip. The cells and medium were pooled and titered on PK15 cells.

### Detecting axon-mediated infection of neuronal cell bodies

Two weeks postplating, 330 μl of neuron medium was placed in the somal compartment. About 10^6^ pfu of virus in 270 μl neuron medium was subsequently added to axonal compartment and incubated for 1 hour to allow virus entry. The viral inoculum was then removed and replaced with 270 μl of neuron medium, with the volume difference between the two compartments maintained. The chambers were incubated in a humidified 37°C incubator until the appropriate time after infection. For titer determination, neurons and medium were harvested from the somal compartment and titered on PK15 cells.

### Indirect immunofluorescence assays

At the appropriate time after infection of the somal compartment, the somal and axonal compartments were washed once with phosphate-buffered saline (PBS) and fixed with 4% paraformaldehyde for 10 min. After removal of the fixative, the cells were washed two more times with PBS. Cells were then incubated with PBS containing 3% bovine serum albumin (BSA) and 0.5% triton for 10 min, and PBS containing 3% BSA for 30 min before the addition of primary antibodies for 1 hour. After 1 hour, the primary antibodies were removed and the sample was washed two times with PBS containing 3% BSA and 0.5% saponin. Next, secondary antibodies were applied for 1 hour. All antibodies were diluted in PBS containing 3% BSA and 0.5% saponin. After 1 hour, the secondary antibodies were removed and the sample was washed two times with PBS containing 3% BSA and 0.5% saponin. After washing once with PBS, and once with dH2O, Aqua poly/mount (Polysciences) was added to the somal and axonal compartments. The Aqua poly/mount (Polysciences) was air dried for 24 h prior to imaging. Wide-field epifluorescence microscopy was performed using a Nikon Eclipse TE 2000-U microscope equipped with a Cooke SensiCam High Performance camera. Images were acquired using IP lab software (Scanalytics Inc.).

### Confocal microscopy and live imaging

Fixed samples were imaged with a Perkin-Elmer (Wellesley, California, United States) RS3 spinning disk confocal system side-mounted on a TE2000-S Nikon Eclipse microscope (Tokyo, Japan) with an Argon/Krypton laser producing excitation lines of 488, 568, and 647 nms. Live imaging was performed using a Leica SP5 with an HCX Plan Apochromat 63X 1.3NA glycerin objective. 20 mM HEPES was added to the medium prior to imaging. The chamber was warmed to 37°C employing a DH40i Micro-incubation system (Warner Instrument Corp.) run at constant voltage. Laser lines at 488 and 561 nm were used for simultaneous GFP and RFP excitation, with emissions from 495 to 553 nm and 587 to 700 nm collected for GFP and RFP respectively. Images were acquired employing a 2.7-Airy-unit detector pinhole and scanning at a speed of 1,000 Hz in a bidirectional mode. A 3-by-3 kernel median filter was applied to the data post-acquisition. For tracking of PRV181 particles, over 80 recordings were acquired from three independent experiments at 11–14 hpi, with each recording typically lasting 2 min and having a field of view of 50 μm. Average velocities were calculated from periods of uninterrupted motion in a given direction. All figures were assembled in Adobe Photoshop 7.0.1.

## Results

### The Microfluidic Culture System

The microfluidic chamber consists of a molded elastomeric polymer placed on a glass coverslip (Fig. 1A). A physical barrier with embedded microgrooves connects the somal and axonal compartments. Microgrooves that connect the compartments allow the passage of neurites to the axonal compartment, but not of cell bodies. Neurite growth is very robust, and typically, neurites extend into the axonal compartment within 5 days in culture. After 9 days in culture, cell bodies in the chamber are readily labeled with the somatodendritic marker nonphosphorylated neurofilament H while neurites are labeled with the axonal marker phosphorylated neurofilament H (Fig. 1B). Only axons were detected in the axonal compartment.

To demonstrate that virus can be restricted to one compartment of the chamber, we cultured PK15 cells in both the somal and axonal compartments of 10 separate chambers. After allowing the cells to settle and attach for 24 h, we established a volume difference of 30 μl between the two compartments, with the somal compartment containing the lesser volume. The hydrostatic pressure difference counteracts diffusion [39], [40]. PK15 cells in the somal compartment were then infected with GS443, a PRV strain that expresses the fusion protein GFP-VP26 (green fluorescent protein fused to the PRV capsid protein VP26). Cells in both compartments were imaged at 24 and 48 h postinfection. Infected cells were detected by green fluorescence emitted from GFP-VP26. In all 10 chambers, only PK15 cells in the somal compartment were infected (data not shown). None of the PK15 cells in the axonal compartment were infected as demonstrated by the lack of green fluorescent signal.

### Spread of infection from neuron cell bodies to detector cells in the axonal compartment requires intact axons

**Figure 2:**
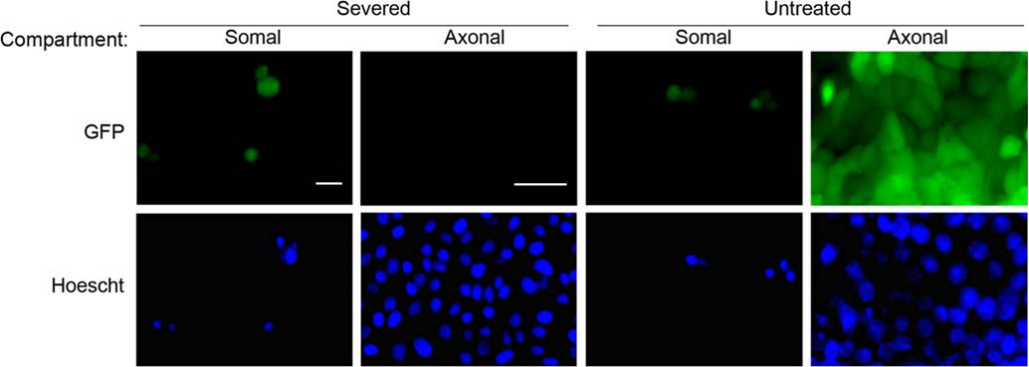
Neuron-to-cell spread of PRV requires intact axons. PK15 cells were plated in the axonal compartment. Neurons in the somal compartment were then infected at a high MOI with PRV 151, a strain that expresses GFP. At 1 h postinfection, all axons in the axonal compartment were either severed or left untreated as a control. Cells were fixed at 24 h postinfection and imaged using wide-field epifluorescence microscopy. Bars: 50 μm.

We used the chamber to study spread of infection from neurons to detector cells. After neurons have matured for 2 weeks and axons have extended to the axonal compartment, we plated PK15 cells in the axonal compartment (*n* = 6 chambers). At 24 h postplating, complete monolayers formed in the axonal compartment. Neurons in the somal compartment were infected at a high MOI with PRV 151, a strain that expresses diffusible GFP. For half the samples, we severed the axons in the axonal compartment by vacuum aspiration 1 hour postinfection upon removal of viral inoculum [30]. Due to the high fluidic resistance of the microgrooves, the cell bodies in the somal compartment remained undisturbed. We left the remaining samples untreated. After 24 h postinfection, we detected transmission of infection by GFP expression. Severing of axons from their cell bodies blocked neuron-to-cell spread of infection, indicating that transmission of infection requires the presence of intact axons (Fig. 2). These results also suggest that spread of infection to the axonal compartment does not occur through leakage of extracellular virus particles from the somal compartment.

### PRV Bartha is defective in neuron-to-cell spread of infection

**Figure 3:**
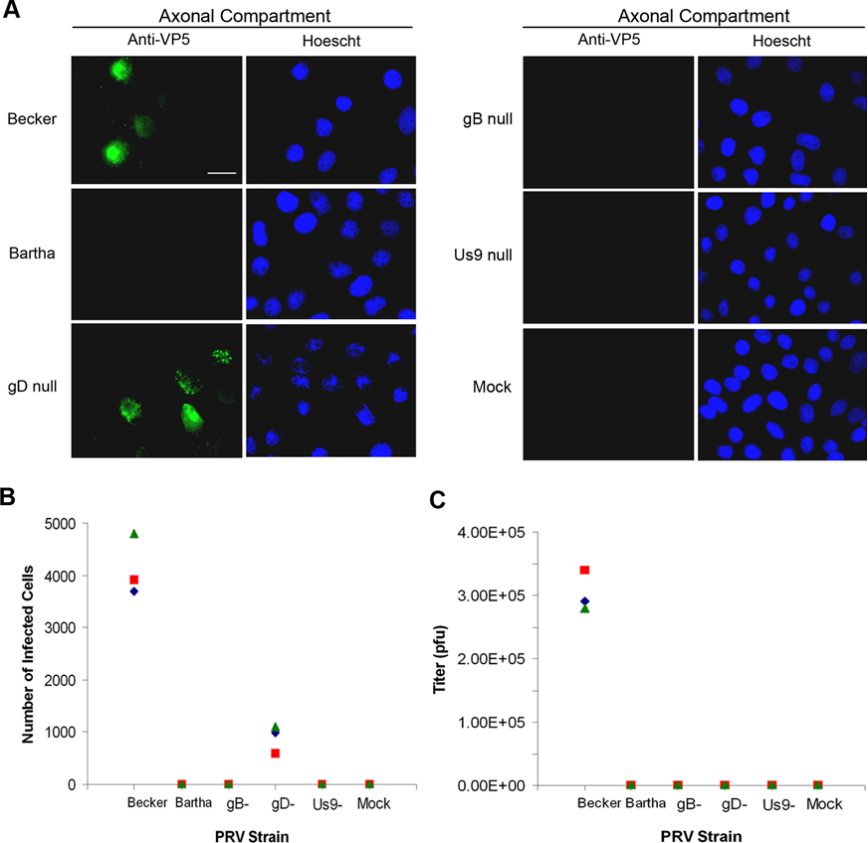
Assaying for viral proteins required for neuron-to-cell spread of infection. Neurons in the somal compartment were infected at high MOI with PRV Becker, PRV Bartha, GS442 (a complemented gD-null virus that expresses GFP), HF22A (a complemented gB-null virus), or PRV 160 (Us9-null). Three chambers were used for each PRV strain. (A) Epifluorescent images of PK15 cells in the axonal compartment fixed and stained with antibodies against the VP5 capsid protein and the Hoechst nuclear dye at 20 h postinfection. Neurons in the somal compartment are not shown. (B) Quantitation of infected cells in the axonal compartment. The number of cells positive for VP5 staining was determined for each chamber by calculating the total area of infected monolayer. (C) Quantitation of infectious particles in the axonal compartment. Medium and cells in the axonal compartment were harvested at 20 h postinfection, and the titer was determined on PK15 cells. Bar: 25 μm.

The attenuated vaccine strain PRV Bartha is defective in anterograde spread from presynaptic to postsynaptic neurons in many animal models and culture systems [1], [16], [41]. This defect is primarily due to a small deletion that results in partial or complete removal of the gE, gI, and Us9 coding sequences [32], [42]–[44]. However, PRV Bartha can spread in the retrograde direction and is widely used to trace neuronal circuits [45]. We tested whether PRV Bartha exhibits the same defect in neuron-to-cell spread of infection (anterograde spread) in our *in vitro* chamber as observed *in vivo*. We infected cell bodies in the somal compartment with PRV Becker or PRV Bartha, in parallel. After 20 h, neurons and cells in the chamber were fixed and stained with antibody against the major capsid protein VP5 and Hoechst stain to detect nuclei. We measured the degree of spread by calculating the number of VP5-positive cells in the axonal compartment from the area of infected monolayer (*n* = 3 chambers) (Fig. 3A). As expected, PRV Becker spread from neurons in the somal compartment to cells in the axonal compartment. In contrast, PRV Bartha exhibited a complete defect in neuron-to-cell spread as no infected cells were detected in the axonal compartment (Fig. 3B).

In addition, we determined the yield of infectious particles as a sensitive measure of the degree of infection. PK15 cells in the axonal compartment were harvested at 20 h postinfection and titered. We detected plaques only for PRV Becker-infected chambers and not for PRV Bartha-infected chambers (Fig. 3C). Hence, our results recapitulate the anterograde spread defect of PRV Bartha observed in animal models.

### Neuron-to-cell spread of infection requires gB and Us9 but not gD

We then tested for viral envelope proteins that were necessary for mediating neuron-to-cell spread. First, we determined whether neuron-to-cell spread of infection occurs via a neuron-cell interaction or by infectious free virions. The viral glycoprotein gD is required for entry and fusion of extracellular virus to cells, but is dispensable for direct cell-to-cell spread *in vitro* and *in vivo* [16], [41], [46]–[49]. We infected cell bodies in the somal compartment with PRV GS442, a gD null mutant that expresses GFP (*n* = 3 chambers). We produced infectious PRV GS442 by growing virus stocks on a gD-expressing cell line. Complemented viruses can infect once, but the resulting progeny lack gD, and hence these gD null particles cannot infect extracellularly. Using anti-VP5 and Hoescht staining, we could readily detect infected cells in the axonal compartment (Fig. 3A). We scored the number of infected cells in the chamber infected with the gD mutant by determining the total area of infected monolayer. We found that the gD null infection produced a 4-fold decrease in the number of infected cells compared to a PRV Becker infection (Fig. 3B). This is not surprising as extracellular virus released from PRV Becker-infected PK15 cells could spread extensively to non-neighboring cells in the compartment while progeny of PRV GS442 could only spread between neighboring cells. We then harvested cells in the axonal compartment from the gD null infection and determined the titer. We did not detect any plaques (Fig. 3C), indicating that there were no gD-positive revertants in our gD mutant stocks. We conclude that neuron-to-cell spread does not require gD.

The essential viral glycoprotein gB is required for spread of infection via cell-to-cell spread as well as by extracellular particles *in vitro* and *in vivo* [16], [48], [49]. We confirmed that gB null mutants are unable to spread from neurons to cells in our chamber. We infected cell bodies in the somal compartment with a complemented PRV gB null virus (PRV HF22A) and scored for infected cells in the axonal compartment using anti-VP5 and Hoescht staining (*n* = 3 chambers). Like a complemented gD null mutant, a complemented gB null mutant can only infect once as its progeny do not contain gB and are noninfectious. We did not detect any cells in the axonal compartment that stained positive for VP5 (Fig. 3A and B). Furthermore, cells in the axonal compartment failed to produce any plaques (Fig. 3C). These assays confirm that gB is necessary for neuron-to-cell spread.

Finally, we show that the viral membrane protein Us9 is required for neuron-to-cell spread. Us9 is necessary for targeting viral structural proteins to the axon [50], [51]. Us9 null mutants are defective in anterograde spread of infection in the rat visual system and in cultured neurons [41], [50], [52]. Upon infecting neuronal cell bodies with PRV 160, a Us9 null mutant, we failed to detect VP5 staining in cells of the axonal compartment (*n* = 3 chambers) (Fig. 3A and B). Our plaque assay confirmed that there were no infectious particles in the axonal compartment at 20 h postinfection (Fig. 3C).

### Axon-mediated infection can occur in the retrograde direction

**Figure 4:**
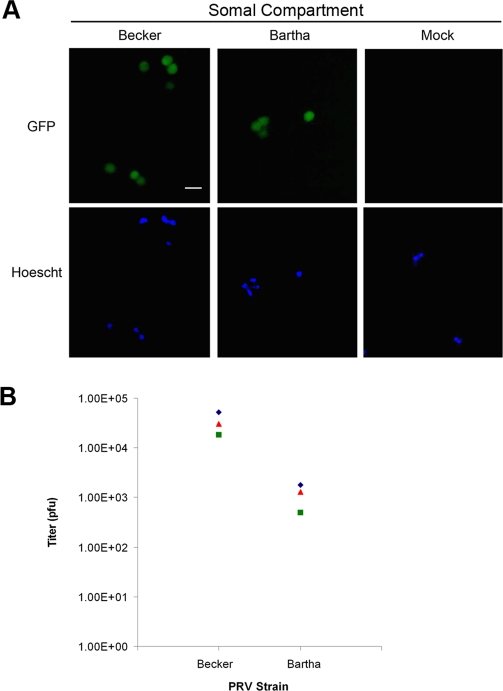
PRV Bartha has a slight defect in retrograde spread of infection to cell bodies. A viral inoculum of PRV151 (a Becker recombinant expressing GFP) or PRV152 (a Bartha recombinant expressing GFP) was incubated in the axonal compartment for 1 h before being replaced with neuronal medium. Three chambers were used for each PRV strain. (A) Epifluorescent and Hoechst images of cell bodies in the somal compartment fixed at 20 h postinfection. (B) Quantitation of infectious particles in the somal compartment. Medium and cells in the somal compartment were harvested at 20 h postinfection and titered on PK15 cells. Bar: 50 μm.

Our previous experiments recapitulated viral spread in the anterograde direction. We next used the chamber to study axon-mediated infection of cell bodies. We added 10^6^ pfu of PRV Becker (PRV151) or PRV Bartha (PRV152) to the axonal compartment without plating any detector cells (*n* = 3 chambers). PRV151 and PRV152 are each recombinant strains expressing freely diffusible GFP. At 24 h postinfection, we identified infected cell bodies by fluorescence emission. Both PRV Becker and PRV Bartha infected cell bodies in the somal compartment, suggesting that virus entered and traveled through axons in the axonal compartment to infect cell bodies (Fig. 4A). However PRV Bartha consistently yielded lower titers in the somal compartment than PRV Becker (Fig. 4B). This result is consistent with observations that PRV Bartha spreads slower than PRV Becker in animal models and *in vitro* [41], [53], [54].

### Live imaging of capsid and tegument transport

To demonstrate the value of the microfluidic chamber to study the transport of virion components during viral egress inside the chamber, we used PRV181, a recombinant virus expressing two fluorescent proteins: GFP fused with the outer tegument protein VP22 and mRFP fused to capsid protein VP26 [35]. To ensure that the fluorescent fusion proteins do not interfere with axonal transport, we assayed for neuron-to-cell spread of PRV181 and compared it to that of PRV Becker (*n* = 3 chambers). After infecting cell bodies in the somal compartment for 24 h, we harvested and titered cells and medium from the somal and axonal compartments. Since PRV181 has no replication defect in PK15 cells [35], lower titers would indicate a kinetic defect in spread from neurons to PK15 cells. We found that PRV Becker and PRV181 produced comparable titers in both somal and axonal compartments (*t* test; alpha = 0.05) (Table 1). Hence, we conclude that PRV181 has no obvious defect in anterograde transport and proceeded to use the virus for live-cell imaging.

**Table 1:** Titers of PRV Becker and PRV181 in the axonal compartment upon neuron-to-cell spread of infection.

	Somal Compartment		Axonal Compartment	
	PRV Becker	PRV 181	PRV Becker	PRV 181
*n*	3	3	3	3
Titer^1^ (×10^6^ pfu)	15.3±4.1	8.7±7.2	10.3±8.9	6.2±4.4

1 Average±standard error of the mean.

**Figure 5:**
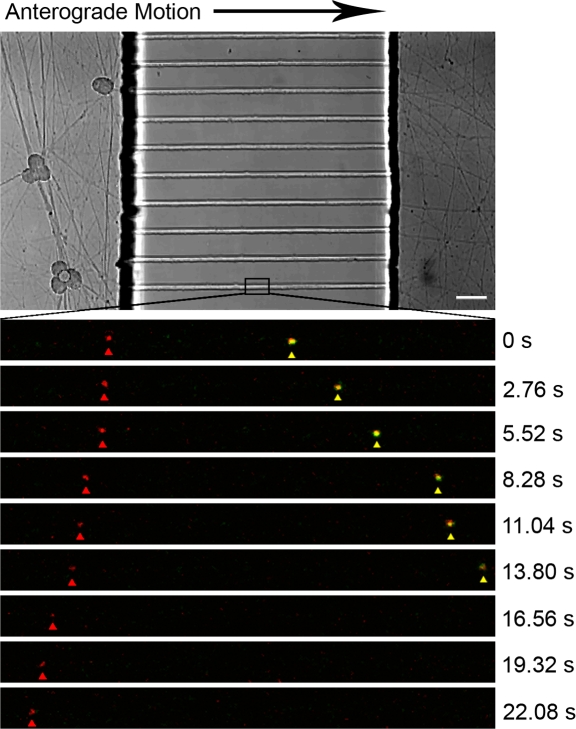
Live imaging of capsid and VP22 transport dynamics inside the microfluidic chamber. Cell bodies in the somal compartment were infected with PRV 181, a recombinant expressing an mRFP-VP26 (capsid) fusion and a GFP-VP22 (outer tegument) fusion. Time-lapse images were taken of axons within the microgrooves at 11–14 h postinfection using the Leica SP5 confocal microscope. A typical region of interest is outlined in the brightfield image. Zoom images show an mRFP punctum moving in the retrograde direction (red arrow) and an mRFP punctum co-transporting with GFP-VP22 punctum in anterograde motion (yellow arrow). For a complete time-lapse recording, see Movie S1 in the supporting information. Bar: 50 μm.

**Figure 6:**
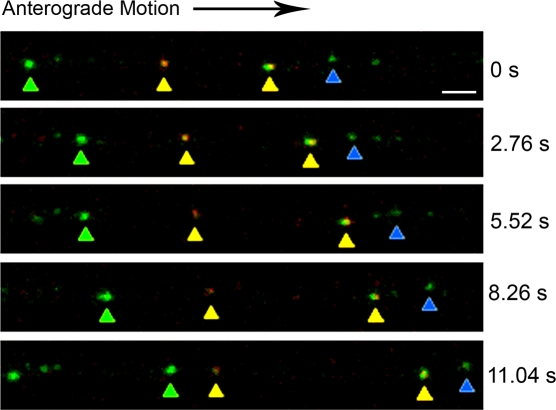
Imaging transport of light and heavy particles inside the microfluidic chamber. Cell bodies in the somal compartment were infected with PRV 181, a recombinant expressing an mRFP-VP26 (capsid) fusion and a GFP-VP22 (outer tegument) fusion. Time-lapse images were taken of axons within the microgrooves at 11–14 h postinfection using the Leica SP5 confocal microscope. Green puncta of light particles containing GFP-VP22 without mRFP-VP26 (green and blue arrows) and yellow punta of heavy particles containing both GFP-VP22 and mRFP-VP26 (yellow and orange arrows) were seen moving in the anterograde direction. Both light and heavy particles displayed heterogeneity in GFP fluorescence. For a complete time-lapse recording, see Movie S2 in the supporting information. Bar: 2 μm.

**Figure 7:**
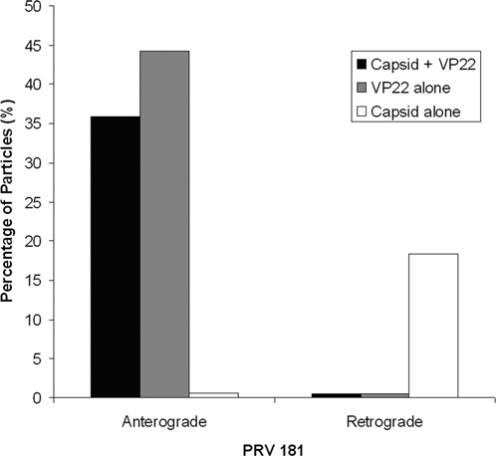
Transport direction of capsids and VP22 in axons during viral egress. Cell bodies in the somal compartment were infected with PRV 181, a recombinant expressing an mRFP-capsid fusion and a GFP-VP22 fusion. Viral particles traveling in axons within the microgrooves were tracked from 11–14 h postinfection. The fraction of capsids transported with (black) or without (white) VP22 and the fraction of VP22 moving in the absence of capsids (grey) are shown as percentages of the total particles tracked.

We infected neurons in the somal compartment with PRV181 and imaged progeny viral particles within axons inside the microgrooves at 11 to 14 hpi, the peak time for capsid transport to axon terminals [4]. We did not plate any detector cells in the axonal compartment. The microgrooves define transport direction, allowing us to clearly distinguish anterograde from retrograde motion. We observed three classes of puncta: red, green and yellow (Fig. 5 and 6; Supporting Information Movie S1 and S2). The green puncta most likely are light particles containing GFP-VP22 without capsid, the yellow puncta are heavy particles with both GFP-VP22 and mRFP-VP26, and the red puncta are capsids without outer tegument, although small quantities of green fluorescence may be below our detection limit. Both heavy and light particles displayed heterogeneity of GFP fluorescence (Fig. 6; Supporting Information Movie S2), as has been observed for extracellular virions [35]. mRFP puncta (capsid structures) traveled predominantly in the anterograde direction, although processive retrograde transport also occurred at a lower frequency, as described previously [4]. We observed that almost all mRFP puncta traveling in the anterograde direction were associated with GFP-VP22 signal: 238 out of 242 mRFP puncta (98.3%) emitted GFP fluorescence (Fig. 7). In addition, we observed that GFP-VP22 puncta frequently traveled in the anterograde direction in the absence of any detectable mRFP signal (Fig. 7). In contrast, mRFP puncta moving in the retrograde direction were generally not coupled with green fluorescence (122 out of 125 total) (Fig. 5 and 7; Supporting Information Movie S1). GFP-VP22 puncta almost never traveled in the retrograde direction (3 out of 297 total). Hence, processive retrograde transport of capsid structures occurred in the absence of VP22 (outer tegument) during viral egress in axons.

**Figure 8:**
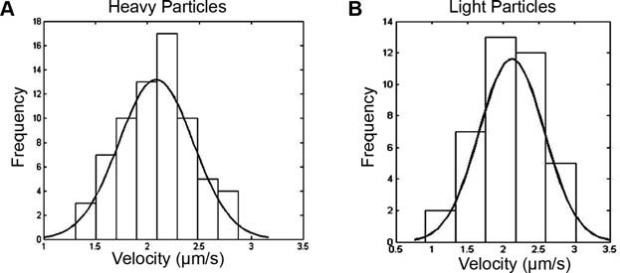
Distribution of transport velocities for heavy and light particles. Histogram of anterograde transport velocities of individual heavy (*n* = 69) (A) and light (*n* = 43) (B) particles resulting from infections of cell bodies in the somal compartment with PRV 181. The best-fit Gaussian curve for each sample is shown.

To compare the transport dynamics of light and heavy particles, we tracked individual VP26 (heavy) and VP22 (light) fluorescent puncta as they traveled inside the microgrooves of the chamber. The velocity distributions for both light (*n* = 43) and heavy (*n* = 69) particles can be modeled with a Gaussian distribution (Shapiro-Wilk test for normality; p-value>0.6) (Fig. 8). Heavy particles moved with an average velocity of 2.17±0.04 μm/s while light particles traveled at 2.13±0.07 μm/s (Table 2). The differences between the two velocities were not statistically significant (*t* test; alpha = 0.05). The average velocity of capsids moving in the retrograde direction was 0.70±0.04 μm/s (*n* = 39) (Table 2). Previous studies in chick dorsal root sensory neurons have reported a similar anterograde capsid velocity and a retrograde velocity twice that obtained in this study [3]. This apparent discrepancy may be due to differences in the type of neuron used.

**Table 2:** Transport velocities of capsids and VP22 during viral egress.

	Anterograde		Retrograde
	Capsid+VP22	VP22 alone	Capsid alone
*n*	69	43	39
Velocity^1^ (μm/s)	2.17±0.04	2.13±0.07	0.70±0.04

1 Average±standard error of the mean.

## Discussion

The microfluidic chamber provides a facile platform for studying neuron-to-cell transmission of infection and viral transport *in vitro*. The chamber is fabricated using soft lithography, resulting in reproducible and high-throughput experiments. It uses hydrostatic pressure to fluidically isolate virus to one compartment of the chamber. This feature eliminates the need to apply a silicon grease layer. The chamber is compatible with classical virology methods such as titering and immunofluorescence assays for assessing neuron-to-cell spread. Furthermore, it is suited for high-resolution live imaging of viral axonal transport.

In this report, we validated the microfluidic chamber system for analysis of neuron-to-cell spread of alpha-herpesvirus infection from SCG neurons to epithelial cells. This spread is axon-mediated, and requires the presence of the gE, gI, and Us9 proteins, but not gD. In addition, gB-mediated fusion is required. By these criteria, our system faithfully recapitulates *in vivo* anterograde spread of infection from presynaptic neurons to postsynaptic cells in close proximity. Furthermore, the chamber can be used to study retrograde spread of infection to cell bodies.

The microfluidic chamber offers technical advantages over previous Campenot chambers that have been developed to study viral spread from neurons to cells [9]–[12], [14], [15]. First, axons grow through embedded microgrooves in the microfluidic chamber and hence are not disturbed throughout the experiment. Previous chambers require silicone grease or agarose to be applied on top of axons as a viral diffusion barrier. An imperfect grease seal often led to leakiness and disruption of neurites. Second, the microgrooves allow for high efficiency of axonal isolation in the axonal compartment. Other chamber systems utilize axon guidance grooves etched on the culturing surface to encourage axon growth under the chamber, which may result in low efficiency of axon penetration and incompatibility with live-cell imaging. Third, the microfluidic chamber can be easily adapted to isolate proximal and distal segments of the axon by varying the length of the microgrooves. Fourth, the directionality of the microgrooves defines retrograde and anterograde transport during time-lapse imaging. A large number of axons can be analyzed in a given chamber, facilitating quantification of transport.

Using live-cell laser scanning microscopy, we imaged capsid and tegument protein transport in axons within the microgrooves of the chamber during viral egress. We isolate virus to the somal compartment, hence separating the site of infection from the site of imaging. This method removes potential ambiguities introduced by uptake of input virions at axon termini and clarifies transport direction.

We observed that a large proportion of GFP-VP22 puncta traveled in the anterograde direction independent of capsid puncta (54.6% of total). These structures are likely to be light particles and have a velocity distribution similar to that of heavy particles. This observation suggests that capsid structures do not influence the kinetics of anterograde transport. Rather, both heavy and light particles may be carried by host transport vesicles targeted to the axon terminal. Alternatively, a virus protein may recruit kinesin motors to transport viral particles in a capsid-independent manner. The Gaussian velocity distribution indicates that a single class of kinesin motor is likely responsible for anterograde transport of both light and heavy particles. The exact nature of these light particles remains to be determined, but they likely contain tegument and envelope proteins [55].

We have demonstrated that the microfluidic chamber provides a unique tool to study the spread of alphaherpesviruses through time-lapse imaging of axonal transport. There has been a controversy regarding whether herpesvirus components enter the axon as a complete virion, or as subassemblies, with membrane proteins, tegument, and capsids moving separately into axons [11], [12], [56]–[64]. Many of these studies employed imaging of fixed and stained neurons and were thus unable to reflect transport dynamics. In previous live-cell studies, the authors were unable to completely rule out the possibility that the viral particles imaged resulted from endocytosis of virions from the input inoculum [59], [64]. Using the microfluidic chamber, we can unambiguously determine the nature of progeny virions targeted to axons and begin to elucidate the effectors of viral transport.

Importantly, the chamber is adaptable for culturing other types of neurons, including central nervous system neurons, and for patterned cell culture [39]. Culturing of patterned cells inside the microfluidic device allows for the creation of neuronal circuits, potentially enabling us to study circuit-specific spread of alpha-herpesviruses *in vitro*. The chamber is moreover amenable to studying other neurotropic viruses and infectious agents.

## Supporting Information

Movie S1 Time-lapse recording of capsid and VP22 transport dynamics inside the microfluidic chamber. Cell bodies in the somal compartment were infected with PRV 181, a recombinant expressing an mRFP-VP26 (capsid) fusion and a GFP-VP22 (outer tegument) fusion. Time-lapse images were taken of axons within the microgrooves at 11–14 h postinfection using the Leica SP5 confocal microscope. Images were acquired at 4 frames/s.
(5.16 MB AVI)

Movie S2 Time-lapse recording of the transport of light and heavy particles inside the microfluidic chamber. Cell bodies in the somal compartment were infected with PRV 181, a recombinant expressing an mRFP-VP26 (capsid) fusion and a GFP-VP22 (outer tegument) fusion. Time-lapse images were taken of axons within the microgrooves at 11–14 h postinfection using the Leica SP5 confocal microscope. Images were acquired at 4 frames/s.
(8.70 MB AVI)

## References

[1] Enquist LW, Husak PJ, Banfield BW, Smith GA (1998). Infection and spread of alphaherpesviruses in the nervous system.. Adv Virus Res.

[2] Smith GA, Enquist LW (2002). Break ins and break outs: viral interactions with the cytoskeleton of Mammalian cells.. Annu Rev Cell Dev Biol.

[3] Smith GA, Pomeranz L, Gross SP, Enquist LW (2004). Local modulation of plus-end transport targets herpesvirus entry and egress in sensory axons.. Proc Natl Acad Sci U S A.

[4] Smith GA, Gross SP, Enquist LW (2001). Herpesviruses use bidirectional fast-axonal transport to spread in sensory neurons.. Proc Natl Acad Sci U S A.

[5] Tomishima MJ, Smith GA, Enquist LW (2001). Sorting and transport of alpha herpesviruses in axons.. Traffic.

[6] Marchand CF, Schwab ME (1986). Binding, uptake and retrograde axonal transport of herpes virus suis in sympathetic neurons.. Brain Res.

[7] Ziegler RJ, Herman RE (1980). Peripheral infection in culture of rat sensory neurons by herpes simplex virus.. Infect Immun.

[8] Ziegler RJ, Pozos RS (1981). Effects of lectins on peripheral infections by herpes simplex virus of rat sensory neurons in culture.. Infect Immun.

[9] Ch'ng TH, Enquist LW (2006). An in vitro system to study trans-neuronal spread of pseudorabies virus infection.. Vet Microbiol.

[10] Lycke E, Kristensson K, Svennerholm B, Vahlne A, Ziegler R (1984). Uptake and Transport of Herpes Simplex Virus in Neurites of Rat Dorsal Root Ganglia Cells in Culture.. J Gen Virol.

[11] Penfold MET, Armati P, Cunningham AL (1994). Axonal Transport of Herpes Simplex Virions to Epidermal Cells: Evidence for a Specialized Mode of Virus Transport and Assembly.. Proceedings of the National Academy of Sciences.

[12] Holland DJ, Miranda-Saksena M, Boadle RA, Armati P, Cunningham AL (1999). Anterograde Transport of Herpes Simplex Virus Proteins in Axons of Peripheral Human Fetal Neurons: an Immunoelectron Microscopy Study.. J Virol.

[13] Mikloska Z, Cunningham AL (2001). Alpha and Gamma Interferons Inhibit Herpes Simplex Virus Type 1 Infection and Spread in Epidermal Cells after Axonal Transmission.. J Virol.

[14] Mikloska Z, Sanna PP, Cunningham AL (1999). Neutralizing Antibodies Inhibit Axonal Spread of Herpes Simplex Virus Type 1 to Epidermal Cells In Vitro.. J Virol.

[15] De Regge N, Favoreel HW, Geenen K, Nauwynck HJ (2006). A homologous in vitro model to study interactions between alphaherpesviruses and trigeminal ganglion neurons.. Veterinary Microbiology.

[16] Feierbach B, Bisher M, Goodhouse J, Enquist LW (2007). In Vitro Analysis of Transneuronal Spread of an Alphaherpesvirus Infection in Peripheral Nervous System Neurons.. J Virol.

[17] El-Ali J, Sorger PK, Jensen KF (2006). Cells on chips.. Nature.

[18] Breslauer DN, Lee PJ, Lee LP (2006). Microfluidics-based systems biology.. Mol BioSyst.

[19] Andersson H, van den Berg A (2004). Lab-on-Chips for Cellomics.

[20] McDonald JC (2000). Fabrication of microfluidic systems in poly(dimethylsiloxane).. Electrophoresis.

[21] McDonald JC, Whitesides GM (2002). Poly(dimethylsiloxane) as a material for fabricating microfluidic devices.. Accounts Chem Res.

[22] Lin F (2004). Effective neutrophil chemotaxis is strongly influenced by mean IL-8 concentration.. Biochem Biophys Res Commun.

[23] Wang SJ, Saadi W, Lin F, Minh-Canh Nguyen C, Jeon NL (2004). Differential effects of EGF gradient profiles on MDA-MB-231 breast cancer cell chemotaxis.. Exp Cell Res.

[24] Irimia D (2006). Microfluidic system for measuring neutrophil migratory responses to fast switches of chemical gradients.. Lab Chip.

[25] Taylor AM, Rhee SW, Jeon NL (2006). Microfluidic chambers for cell migration and neuroscience research.. Methods Mol Biol.

[26] Saadi W, Wang SJ, Lin F, Jeon NL (2006). A parallel-gradient microfluidic chamber for quantitative analysis of breast cancer cell chemotaxis.. Biomed Microdevices.

[27] Tourovskaia A, Figueroa-Masot X, Folch A (2005). Differentiation-on-a-chip: a microfluidic platform for long-term cell culture studies.. Lab Chip.

[28] Taylor AM (2003). Microfluidic multicompartment device for neuroscience research.. Langmuir.

[29] Taylor AM (2005). A microfluidic culture platform for CNS axonal injury, regeneration and transport.. Nat Methods.

[30] Park JW, Vahidi B, Taylor AM, Rhee SW, Jeon NL (2006). Microfluidic culture platform for neuroscience research.. Nat Protocols.

[31] Platt KB, Mare CJ, Hinz PN (1979). Differentiation of vaccine strains and field isolates of pseudorabies (Aujeszky's disease) virus: thermal sensitivity and rabbit virulence markers.. Arch Virol.

[32] Lomniczi B, Blankenship ML, Ben-Porat T (1984). Deletions in the genomes of pseudorabies virus vaccine strains and existence of four isomers of the genomes.. J Virol.

[33] Demmin GL, Clase AC, Randall JA, Enquist LW, Banfield BW (2001). Insertions in the gG Gene of Pseudorabies Virus Reduce Expression of the Upstream Us3 Protein and Inhibit Cell-to-Cell Spread of Virus Infection.. J Virol.

[34] Jons A, Mettenleiter TC (1997). Green fluorescent protein expressed by recombinant pseudorabies virus as an in vivo marker for viral replication.. Journal of Virological Methods.

[35] del Rio T, Ch'ng TH, Flood EA, Gross SP, Enquist LW (2005). Heterogeneity of a fluorescent tegument component in single pseudorabies virus virions and enveloped axonal assemblies.. J Virol.

[36] Brideau AD, Card JP, Enquist LW (2000). Role of pseudorabies virus Us9, a type II membrane protein, in infection of tissue culture cells and the rat nervous system.. J Virol.

[37] Peeters B, de Wind N, Hooisma M, Wagenaar F, Gielkens A (1992). Pseudorabies virus envelope glycoproteins gp50 and gII are essential for virus penetration, but only gII is involved in membrane fusion.. J Virol.

[38] Ch'ng TH, Flood EA, Enquist LW (2005). Culturing primary and transformed neuronal cells for studying pseudorabies virus infection.. Methods Mol Biol.

[39] Rhee SW, Taylor AM, Tu CH, Cribbs DH, Cotman CW (2005). Patterned cell culture inside microfluidic devices.. Lab Chip.

[40] Taylor AM, Rhee SW, Jeon NL (2006). Microfluidic chambers for cell migration and neuroscience research.. Methods Mol Biol.

[41] Ch'ng TH, Enquist LW (2005). Neuron-to-cell spread of pseudorabies virus in a compartmented neuronal culture system.. J Virol.

[42] Lomniczi B, Watanabe S, Ben-Porat T, Kaplan AS (1984). Genetic basis of the neurovirulence of pseudorabies virus.. J Virol.

[43] Mettenleiter TC, Lukacs N, Rziha HJ (1985). Pseudorabies virus avirulent strains fail to express a major glycoprotein.. J Virol.

[44] Petrovskis EA, Timmins JG, Gierman TM, Post LE (1986). Deletions in vaccine strains of pseudorabies virus and their effect on synthesis of glycoprotein gp63.. J Virol.

[45] Enquist LW, Card JP (2003). Recent advances in the use of neurotropic viruses for circuit analysis.. Current Opinion in Neurobiology.

[46] Babic N, Mettenleiter TC, Flamand A, Ugolini G (1993). Role of essential glycoproteins gII and gp50 in transneuronal transfer of pseudorabies virus from the hypoglossal nerves of mice.. J Virol.

[47] Mulder W, Pol J, Kimman T, Kok G, Priem J (1996). Glycoprotein D-negative pseudorabies virus can spread transneuronally via direct neuron-to-neuron transmission in its natural host, the pig, but not after additional inactivation of gE or gI.. J Virol.

[48] Peeters B, Pol J, Gielkens A, Moormann R (1993). Envelope glycoprotein gp50 of pseudorabies virus is essential for virus entry but is not required for viral spread in mice.. J Virol.

[49] Rauh I, Mettenleiter TC (1991). Pseudorabies virus glycoproteins gII and gp50 are essential for virus penetration.. J Virol.

[50] Lyman MG, Feierbach B, Curanovic D, Bisher M, Enquist LW (2007). Pseudorabies Virus Us9 Directs Axonal Sorting of Viral Capsids.. J Virol.

[51] Tomishima MJ, Enquist LW (2001). A conserved {alpha}-herpesvirus protein necessary for axonal localization of viral membrane proteins.. J Cell Biol.

[52] Brideau AD, Card JP, Enquist LW (2000). Role of Pseudorabies Virus Us9, a Type II Membrane Protein, in Infection of Tissue Culture Cells and the Rat Nervous System.. J Virol.

[53] Card JP, Whealy ME, Robbins AK, Moore RY, Enquist LW (1991). Two [alpha]-herpesvirus strains are transported differentially in the rodent visual system.. Neuron.

[54] Yang M, Card JP, Tirabassi RS, Miselis RR, Enquist LW (1999). Retrograde, Transneuronal Spread of Pseudorabies Virus in Defined Neuronal Circuitry of the Rat Brain Is Facilitated by gE Mutations That Reduce Virulence.. J Virol.

[55] McLauchlan J, Rixon FJ (1992). Characterization of enveloped tegument structures (L particles) produced by alphaherpesviruses: integrity of the tegument does not depend on the presence of capsid or envelope.. J Gen Virol.

[56] Tomishima MJ, Enquist LW (2001). A conserved alpha-herpesvirus protein necessary for axonal localization of viral membrane proteins.. Journal of Cell Biology.

[57] Card JP, Rinaman L, Lynn RB, Lee BH, Meade RP (1993). Pseudorabies virus infection of the rat central nervous system: ultrastructural characterization of viral replication, transport, and pathogenesis.. J Neurosci.

[58] Kritas SK, Pensaert MB, Mettenleiter TC (1994). Invasion and spread of single glycoprotein deleted mutants of Aujeszky's disease virus (ADV) in the trigeminal nervous pathway of pigs after intranasal inoculation.. Vet Microbiol.

[59] Antinone SE, Smith GA (2006). Two modes of herpesvirus trafficking in neurons: membrane acquisition directs motion.. J Virol.

[60] Miranda-Saksena M, Armati P, Boadle RA, Holland DJ, Cunningham AL (2000). Anterograde transport of herpes simplex virus type 1 in cultured, dissociated human and rat dorsal root ganglion neurons.. J Virol.

[61] LaVail JH, Tauscher AN, Aghaian E, Harrabi O, Sidhu SS (2003). Axonal transport and sorting of herpes simplex virus components in a mature mouse visual system.. J Virol.

[62] Lavail JH, Tauscher AN, Hicks JW, Harrabi O, Melroe GT (2005). Genetic and molecular in vivo analysis of herpes simplex virus assembly in murine visual system neurons.. J Virol.

[63] Potel C, Kaelin K, Danglot L, Triller A, Vannier C (2003). Herpes simplex virus type 1 glycoprotein B sorting in hippocampal neurons.. J Gen Virol.

[64] Luxton GW, Haverlock S, Coller KE, Antinone SE, Pincetic A (2005). Targeting of herpesvirus capsid transport in axons is coupled to association with specific sets of tegument proteins.. Proc Natl Acad Sci U S A.

